# Synergism between Basic Asp49 and Lys49 Phospholipase A_2_ Myotoxins of Viperid Snake Venom *In Vitro* and *In Vivo*

**DOI:** 10.1371/journal.pone.0109846

**Published:** 2014-10-07

**Authors:** Diana Mora-Obando, Julián Fernández, Cesare Montecucco, José María Gutiérrez, Bruno Lomonte

**Affiliations:** 1 Instituto Clodomiro Picado, Facultad de Microbiología, Universidad de Costa Rica, San José, Costa Rica; 2 Department of Biomedical Sciences, University of Padova, Padova, Italy; University of Texas Southwestern Medical Center, United States of America

## Abstract

Two subtypes of phospholipases A_2_ (PLA_2_s) with the ability to induce myonecrosis, ‘Asp49’ and ‘Lys49’ myotoxins, often coexist in viperid snake venoms. Since the latter lack catalytic activity, two different mechanisms are involved in their myotoxicity. A synergism between Asp49 and Lys49 myotoxins from *Bothrops asper* was previously observed *in vitro*, enhancing Ca^2+^ entry and cell death when acting together upon C2C12 myotubes. These observations are extended for the first time *in vivo*, by demonstrating a clear enhancement of myonecrosis by the combined action of these two toxins in mice. In addition, novel aspects of their synergism were revealed using myotubes. Proportions of Asp49 myotoxin as low as 0.1% of the Lys49 myotoxin are sufficient to enhance cytotoxicity of the latter, but not the opposite. Sublytic amounts of Asp49 myotoxin also enhanced cytotoxicity of a synthetic peptide encompassing the toxic region of Lys49 myotoxin. Asp49 myotoxin rendered myotubes more susceptible to osmotic lysis, whereas Lys49 myotoxin did not. In contrast to myotoxic Asp49 PLA_2_, an acidic non-toxic PLA_2_ from the same venom did not markedly synergize with Lys49 myotoxin, revealing a functional difference between basic and acidic PLA_2_ enzymes. It is suggested that Asp49 myotoxins synergize with Lys49 myotoxins by virtue of their PLA_2_ activity. In addition to the membrane-destabilizing effect of this activity, Asp49 myotoxins may generate anionic patches of hydrolytic reaction products, facilitating electrostatic interactions with Lys49 myotoxins. These data provide new evidence for the evolutionary adaptive value of the two subtypes of PLA_2_ myotoxins acting synergistically in viperid venoms.

## Introduction

Phospholipases A_2_ (PLA_2_s) are widespread enzymes in snake venoms, where they play major toxic roles in the immobilization and/or killing of prey [1], [2]. Among their diverse activities, myotoxicity is a clinically relevant effect which may lead to severe tissue damage and associated sequelae in envenomings [3]–[5]. Two divergent ancestral PLA_2_ genes representing the group I and group II scaffolds, respectively, were recruited and expressed in the venom gland secretions of Elapidae and Viperidae [6]. Through a process of accelerated evolution [7], these genes accumulated mutations that converted their corresponding non-toxic proteins into potent toxins, most notably displaying neurotoxicity and/or myotoxicity. The independent emergence of such toxic activities in these two lineages of advanced snakes illustrates a case of convergent evolution [8], [9]. A growing body of knowledge has been gathered on the characterization of PLA_2_ toxins, but the structural bases for their toxicity and precise modes of action remain only partially understood, thus leaving opened a number of challenging questions [10].

In the venoms of viperid snakes, two subtypes of myotoxic PLA_2_s can be found, commonly referred to as ‘Asp49’ and ‘Lys49’ variants. The latter, first described in the venom of *Agkistrodon piscivorus piscivorus* [11] and then isolated from many viperid venoms [9], present the substitution of Asp49 by Lys49, a critical change in the catalytic center of the molecule which, together with key amino acid substitutions located in the calcium-binding loop, precludes catalysis [12]–[14]. Therefore, in sharp contrast with their Asp49 PLA_2_ counterparts, the Lys49 myotoxins are enzymatically-inactive PLA_2_ homologues, or ‘PLA_2_-like’ proteins [13], [15]–[18].

Notwithstanding their difference in catalytic activity, both Lys49 and Asp49 PLA_2_ variants display myotoxicity *in vivo* [4], [19]–[21]. The Asp49 PLA_2_s depend on their enzymatic activity to induce skeletal muscle damage, since their catalytic inactivation by covalently modifying His48 with *p*-bromophenacyl bromide results in the loss of myotoxicity [22]–[24]. Furthermore, the toxic effects of Asp49 PLA_2_s on myogenic cells in culture can be mimicked by the products of their hydrolytic activity, i.e. fatty acids and lysophospholipids [25], and hydrolysis of muscle phospholipids of the external monolayer of the sarcolemma by these enzymes has been demonstrated in myotubes in culture as well as in injected mouse muscles [26]. On the other hand, the catalytic-independent mechanism by which Lys49 PLA_2_ homologues induce myonecrosis, has been shown to depend on a cluster of amino acids at their C-terminal region which directly affect the integrity of the sarcolemma [9], [18], [27]–[32].

The venom of *Bothrops asper*, the snake species causing the majority of envenomings in Central America [33], contains multiple Asp49 and Lys49 myotoxin isoforms [34] as well as a non-myotoxic, acidic Asp49 PLA_2_ [35]. In a previous study, a synergistic action between purified Asp49 and Lys49 myotoxins was observed *in vitro*, whereby these two proteins induced a more pronounced Ca^2+^ entry and cell death by acting together, rather than individually [25]. The present work extends these observations by exploring for the first time whether the same phenomenon occurs *in vivo*, and characterizes in further detail relevant features of this synergistic action using an *in vitro* model.

## Materials and Methods

### Isolation of phospholipases A_2_ from *Bothrops asper* venom

Snake venom was collected from specimens kept at the Serpentarium of Instituto Clodomiro Picado, under authorization of the University of Costa Rica. Pooled venom of *Bothrops asper* from the Pacific versant of Costa Rica was fractionated as previously described, to obtain myotoxin II (Lys49; UniProt accession P24605; [36], [37]), a mixture of myotoxins I/III (Asp49; P20474; [24], [38]), and an acidic BaspPLA_2_-II (non-myotoxic, Asp49; P86389; [35]). Fractionation steps included ion-exchange chromatography followed by semi-preparative reverse-phase HPLC on a C_8_ support. Purity was assessed by nano-electrospray mass spectrometry in a QTrap-3200 instrument (ABSciex) operated in positive ion-enhanced multicharge mode, as described [24]. The lack of contaminating Asp49 PLA_2_s in the Lys49 myotoxin II preparation was evaluated by assaying PLA_2_ activity using the synthetic substrate 4-nitro-3-octanoyloxybenzoic acid [39]. Conversely, the lack of contaminating Lys49 myotoxins in the basic Asp49 PLA_2_ myotoxin preparation was ascertained by automated N-terminal amino acid sequencing using a PPSQ-33A instrument (Shimadzu Biotech), to confirm the absence of a Leucine signal in the fifth cycle [24].

### Asp49 PLA_2_ myotoxin inactivation by *p*-bromophenacyl bromide

Three mg of the Asp49 myotoxin were dissolved in 1 mL of 0.1 M Tris, 0.7 mM EDTA, pH 8.0 buffer. Then, 125 µL of *p*-bromophenacyl bromide (*p*-BPB; 1.5 mg/mL in ethanol; Sigma Chemical Co.) were added and incubated at room temperature (20–25°C) for 24 h [22]. Excess *p*-BPB and salts were eliminated by RP-HPLC on a semi-preparative C_8_ column, as described [24]. The protein was collected and finally dried by vacuum centrifugation at 45°C. Enzymatic inactivation was determined on the 4-nitro-3-octanoyloxybenzoic acid substrate in comparison to a control sample of the toxin which was processed identically but omitting the *p*-BPB reagent [24].

### Synthetic peptide of *B. asper* myotoxin II

A synthetic peptide from the C-terminal region of *B. asper* myotoxin II, corresponding to the sequence 115–129 (KKYRYYLKPLCKK; p^115–129^), was obtained from a commercial provider (Peptide 2.0, Inc.). The peptide was synthesized with native endings by Fmoc chemistry, and its molecular mass was in agreement with the expected value. Its purity level was at least 95% by RP-HPLC analysis. This 13-mer peptide has been shown to reproduce, albeit with a lower potency, the cytolytic effect of myotoxin II *in vitro* [27], [29].

### Cytotoxic activity

Cytolysis was determined on the murine myogenic cell line C2C12 (ATCC-CRL1772) using a lactate dehydrogenase release assay, as previously described [40]. Cells were grown at subconfluent densities in 25 cm^2^ bottles using Dulbecco’s modified Eagle’s medium supplemented with 10% fetal calf serum (DMEM, 10% FCS), and after detachment by trypsin, they were seeded in 96-well plates for cytotoxicity assays. These were performed either at the myoblast stage in near-confluent cell monolayers, or after their differentiation to fused myotubes in DMEM 1% FCS during 4–6 additional days. In brief, different amounts of toxins, alone or in combination, dissolved in 150 µL of assay medium (DMEM, 1% FCS) were added to the cells immediately after removal of their medium, and incubated for 3 h at 37°C. Then, an aliquot of cell supernatant (60 µL) was collected from each well and the lactacte dehydrogenase (LDH) activity was quantified by a UV kinetic assay (LDH-BR Cromatest, Linear Chemicals). Controls for 0 and 100% cytotoxicity consisted of assay medium, and 0.1% Triton X-100 diluted in assay medium, respectively. All samples were assayed in triplicate wells.

### Myotoxic activity

Myotoxic activity was determined in CD-1 mice of 18 to 20 g of body weight, using five animals per group. These *in vivo* assays were kept to a minimum, and followed protocols authorized by the Institutional Committee for the Use and Care of Animals (CICUA; #132-13), University of Costa Rica. Mice were housed in cages for groups of 4–6, and provided food and water *ad libitum*. Different amounts of the toxins, alone or in combination, dissolved in 50 µL of phosphate-buffered saline (PBS; 0.12 M NaCl, 0.04 M sodium phosphate buffer, pH 7.2), were injected into the gastrocnemius muscle [36]. A control group of mice received an identical injection of PBS. After 3 h, blood was collected from the tip of the tail into a heparinized capillary and centrifuged. The plasma creatine kinase (CK) activity, expressed in U/L, was determined using a UV kinetic assay (CK-Nac, Biocon Diagnostik). Mice were sacrificed by CO_2_ inhalation, at the end of the experiment.

### Statistical analysis

ANOVA was used for the comparison of mean values from more than two groups, followed by Tukey-Kramer tests, with a statistical significance of p<0.05. Calculations were performed with the aid of the Instat (GraphPad) software.

## Results

**Figure 1:**
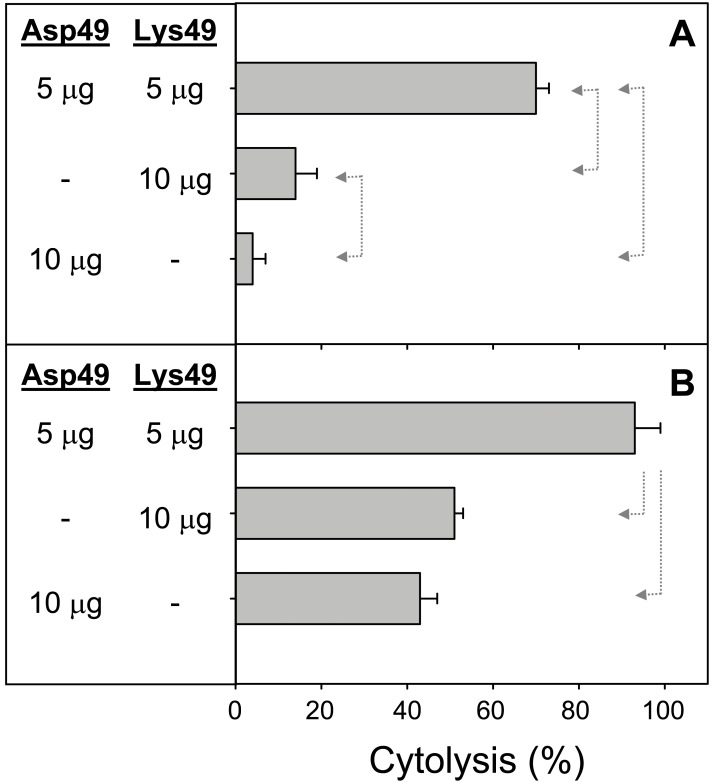
Cytotoxic activity of Asp49 and Lys49 myotoxins from *Bothrops asper*, alone or in combination, upon C2C12 myoblasts (A) or myotubes (B). The indicated amounts of toxins were added in a total volume of 150 µL. Cytolysis was determined by the release of lactate dehydrogenase to the medium 3 h after exposure of the cells to the toxins, as described in Materials and Methods. Reference values of 0 and 100% cytolysis were established using medium or 0.1% Triton X-100 in medium, respectively. Each bar represents mean ± SD of triplicate cell cultures. Statistically significant differences (p<0.05) between two groups are indicated by dotted arrow lines.

**Figure 2:**
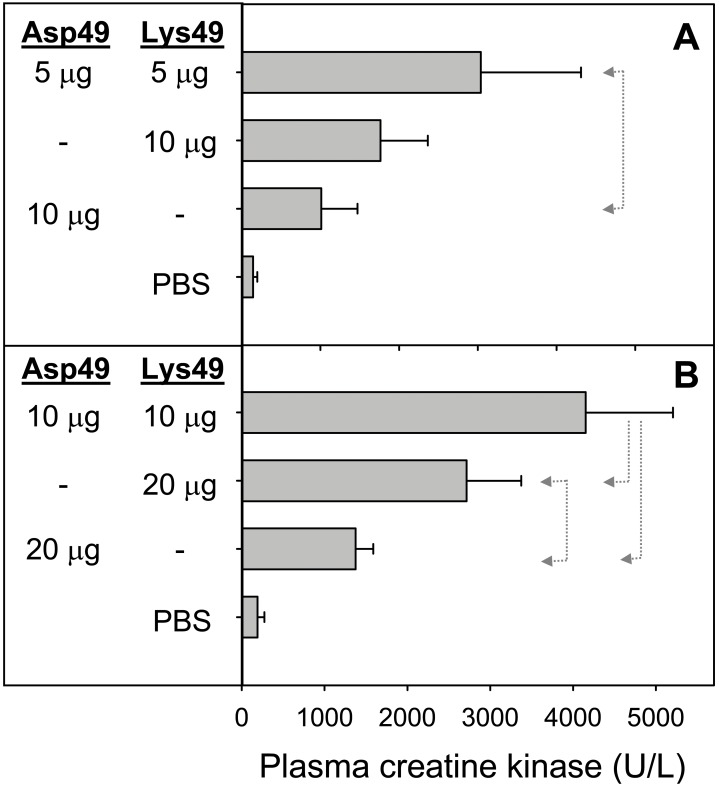
Myotoxic activity of Asp49 and Lys49 myotoxins from *Bothrops asper*, alone or in combination, injected by intramuscular route in CD-1 mice. Plasma creatine kinase activity was determined 3 h after injection of the indicated amounts of toxins. Control mice received only vehicle (PBS). Each bar represents mean ± SD of five animals. Statistically significant differences (p<0.05) between two groups are indicated by dotted arrow lines. Synergistic action is shown at a total dose of 10 µg in (A) or 20 µg in (B), respectively.

The cytolytic effect of Asp49 and Lys49 myotoxins, when added alone or in combination to cultures of the C2C12 cell line at the myoblast or myotube stages, is shown in Fig. 1. A higher effect of these toxins was observed in myotubes than in myoblasts, and this difference was more conspicuous in the case of the Asp49 myotoxin, which was extremely weak against myoblasts (Fig. 1A). In both stages of cell differentiation, the combination of the myotoxins induced a significantly higher cytotoxicity in comparison to the effect of either toxin alone (Fig. 1). The observed effect was clearly synergistic and not just additive. Following these *in vitro* findings, experiments were performed in mice to determine whether synergism also occurs in mature skeletal muscle, under conditions that mimic envenomings. Results revealed a clear enhancement of myotoxicity, as judged by the higher release of creatine kinase from damaged muscle to the plasma, when the Asp49 and Lys49 toxins acted in combination (Fig. 2). When injected individually, the myotoxic effect of the Lys49 myotoxin was significantly higher than that of the Asp49 myotoxin at the dose of 20 µg (Fig. 2B), although at 10 µg (Fig. 2A) this same trend did not reach statistical significance.

**Figure 3:**
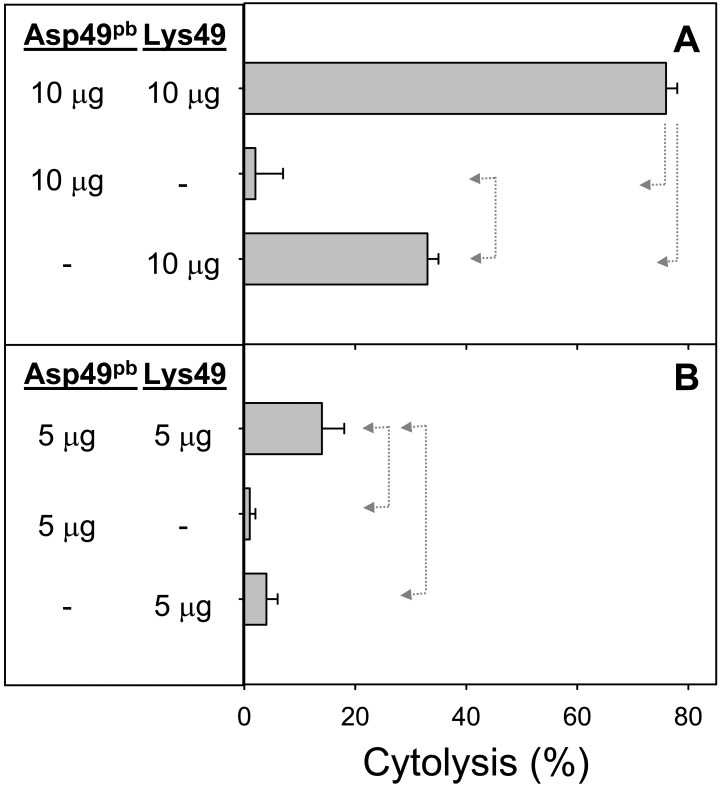
Cytotoxic activity of *p*-bromophenacyl bromide-modified Asp49 myotoxin (Asp49^pb^) and Lys49 myotoxin, alone or in combination at 10 µg (A) or 5 µg (B), upon C2C12 myotubes. The indicated amounts of toxins were added in a total volume of 150 µL. Cytolysis was determined by the release of lactate dehydrogenase to the medium, 3 h after exposure of the cells to the toxins. Each bar represents mean ± SD of triplicate cell cultures. Statistically significant differences (p<0.05) between two groups are indicated by dotted arrow lines.

**Figure 4:**
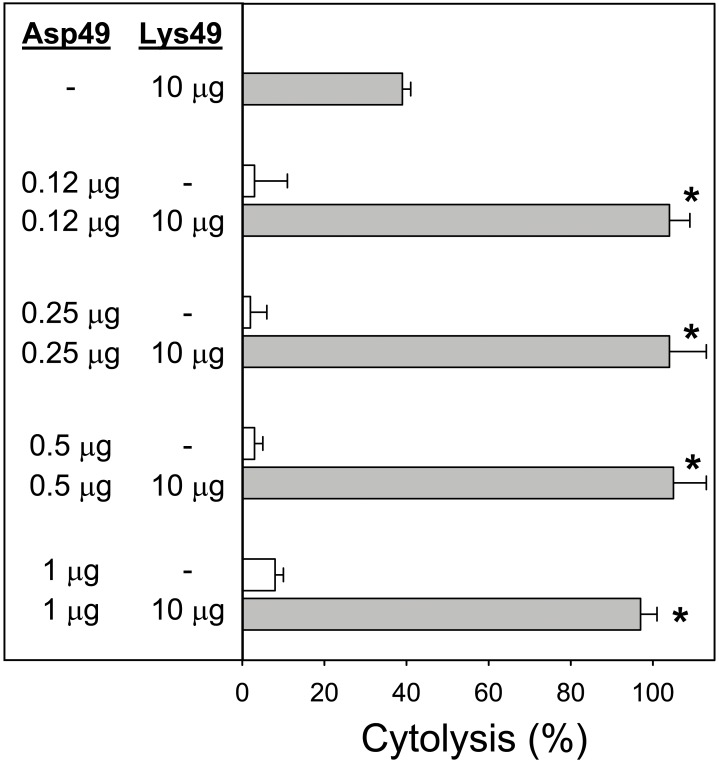
Cytotoxic activity of a fixed amount of Lys49 myotoxin, alone or in combination with low amounts of Asp49 myotoxin, upon C2C12 myotubes. The indicated amounts of toxins were added in a total volume of 150 µL. Cytolysis was determined by the release of lactate dehydrogenase to the medium, 3 h after exposure of the cells to the toxins. Each bar represents mean ± SD of triplicate cell cultures. All values from cultures where the Lys49 myotoxin was added together with Asp49 myotoxin were significantly different (p<0.05) from the value of cultures exposed only to Lys49 myotoxin (indicated by asterisks).

**Figure 5:**
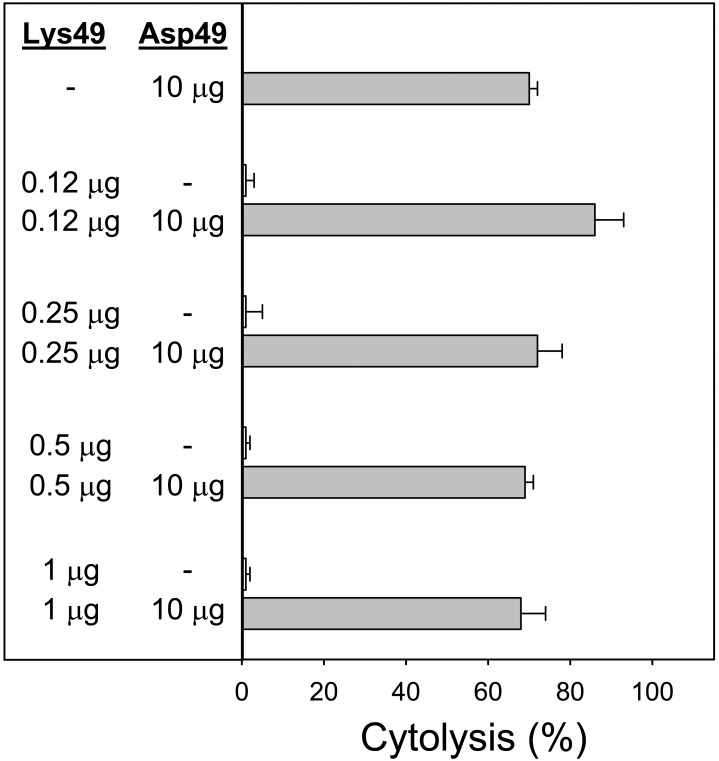
Cytotoxic activity of a fixed amount of Asp49 myotoxin, alone or in combination with low amounts of Lys49 myotoxin, upon C2C12 myotubes. The indicated amounts of toxins were added in a total volume of 150 µL. Cytolysis was determined by the release of lactate dehydrogenase to the medium, 3 h after exposure of the cells to the toxins. Each bar represents mean ± SD of triplicate cell cultures. None of the values from cultures where the Asp49 myotoxin was added together with Lys49 myotoxin were significantly different from the value of cultures exposed only to Asp49 myotoxin.

The role of enzymatic activity of the Asp49 myotoxin in the synergistic effect was studied by using a *p*-BPB-treated enzyme. This protein incorporated a single molecule of the alkylating agent, as confirmed by mass spectrometry, and its catalytic activity was inactivated by 97% in comparison to the untreated enzyme [24]. As shown in Fig. 3, the cytolytic action of the *p*-BPB-treated Asp49 myotoxin alone was negligible, as expected. However, the combined action of this protein and the Lys49 myotoxin caused a significant enhancement of the cytotoxic effect (Fig. 3). Since the *p*-PBP-treated Asp49 protein had a residual enzymatic activity of 3%, further experiments were designed to determine whether the synergistic effect observed in Fig. 3 could be due to this low residual catalytic action or, alternatively, depended on a non-catalytic mechanism of the Asp49 myotoxin. Therefore, low amounts of native Asp49 myotoxin, within a range comparable to the proportion of enzymatically-active protein remaining in the *p*-PBP-treated toxin, were combined with a fixed amount of Lys49 myotoxin (Fig. 4). Results showed that Asp49 myotoxin amounts as low as 0.12 µg (representing 1.2% in proportion to the Lys49 myotoxin), efficiently enhanced the cytotoxicity of the final mixture. Importantly, these low amounts of Asp49 myotoxin were essentially non-toxic when added alone to the myotube cultures (Fig. 4). Further titration of the effect of Asp49 myotoxin in this assay showed that the minimal amount of enzyme capable of inducing synergism was 0.012 µg. The reverse combination, i.e. addition of low quantities of Lys49 myotoxin to a fixed amount of Asp49 myotoxin (Fig. 5) did not enhance toxicity, thus revealing the directionality of the synergistic mechanism.

**Figure 6:**
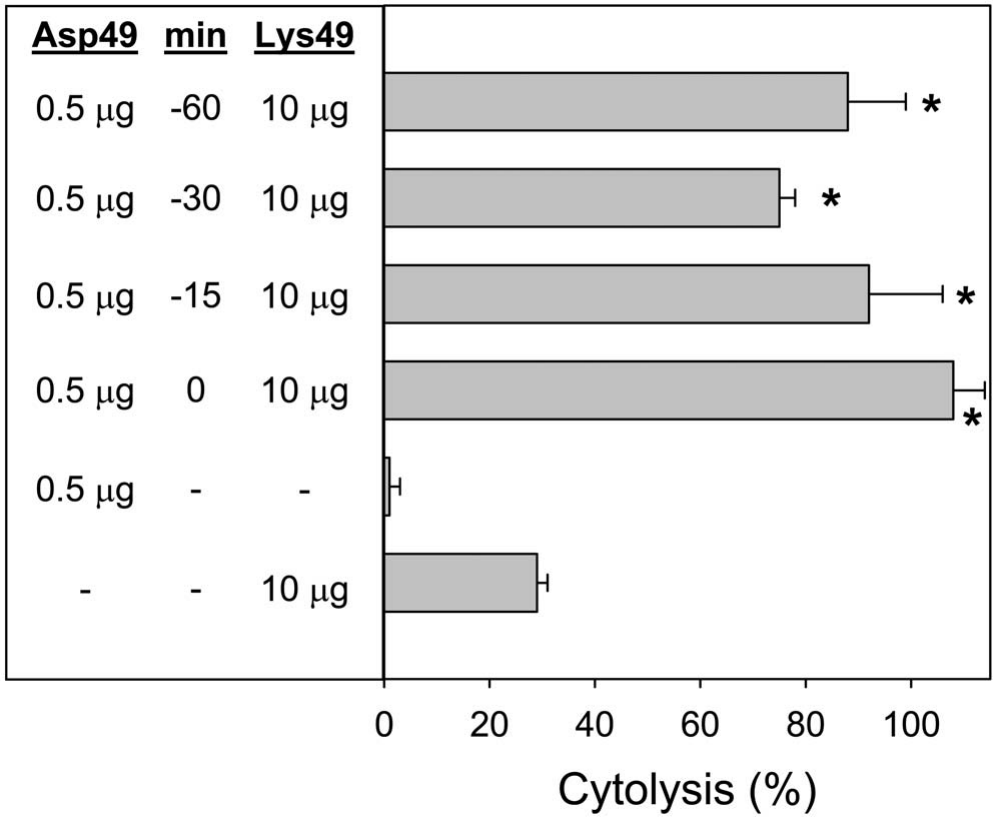
Cytotoxic activity of a fixed amount of Lys49 myotoxin, alone or in combination with a low amount of Asp49 myotoxin. In this experiment, Asp49 myotoxin was first incubated for variable periods of time with C2C12 myotubes, and then washed five times, before the addition of Lys49 myotoxin. The indicated amounts of toxins were added in a total volume of 150 µL. Cytolysis was determined by the release of lactate dehydrogenase to the medium 3 h after exposure of the cells to the toxins. Each bar represents mean ± SD of triplicate cell cultures. All values from cultures where the Lys49 myotoxin was added together with Asp49 myotoxin were significantly different (p<0.05) from the value of cultures exposed only to Lys49 myotoxin (indicated by asterisks).

Since results indicated that the Asp49 myotoxin, even in low amounts, enhanced the toxicity of the Lys49 myotoxin, an experiment was performed to determine whether this synergy was dependent on the time lapse when a low amount of the enzyme was in contact with myotubes, before the addition of the Lys49 myotoxin. The Asp49 enzyme was incubated with the cells for the time periods indicated in Fig. 6 (0, 15, 30, or 60 min) and, after five washings of the cell cultures, the Lys49 myotoxin was added. A significant enhancement in cytotoxicity was recorded at all time periods of cell exposure to the Asp49 myotoxin and, remarkably, even when the Asp49 enzyme was added and the cultures were immediately washed (Fig. 6).

**Figure 7:**
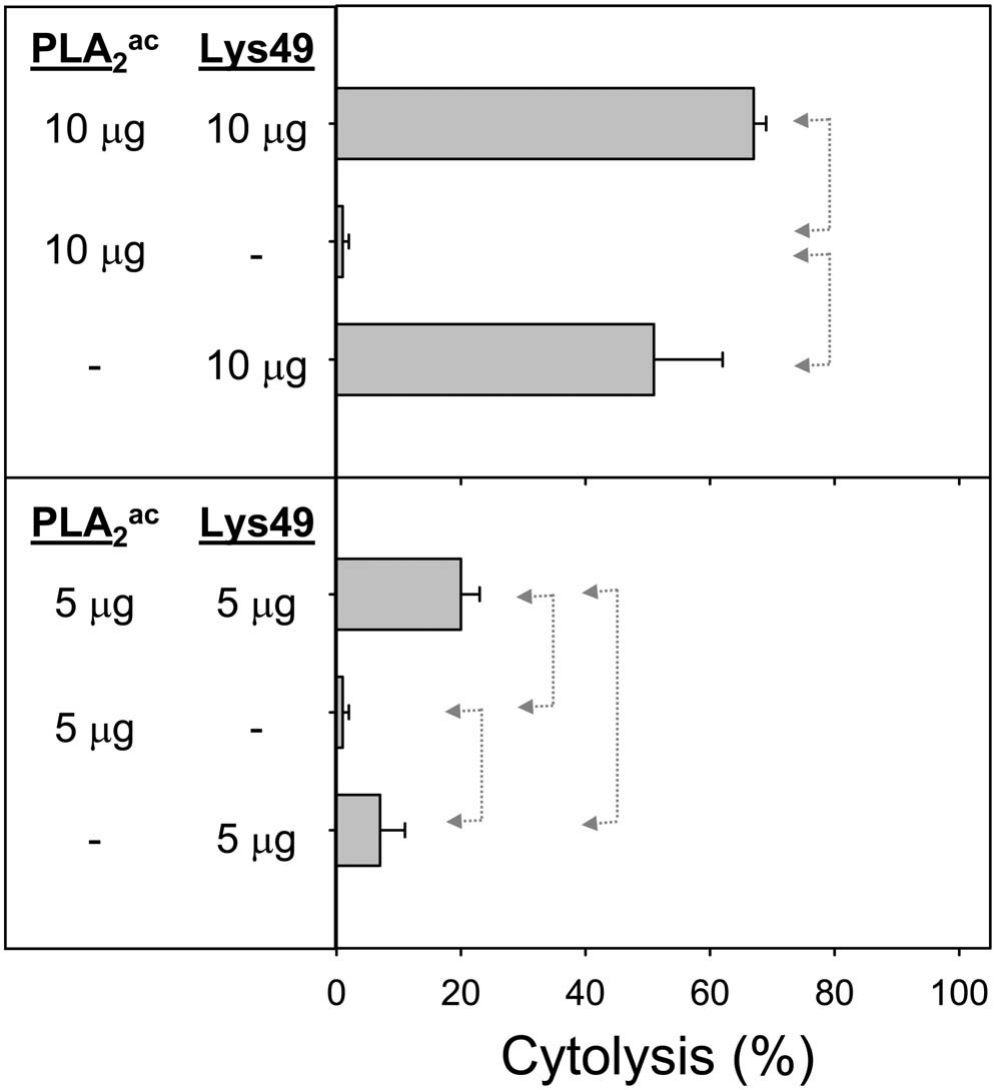
Cytotoxic activity of *Bothrops asper* acidic Asp49 phospholipase A_2_ (PLA_2_^ac^) and Lys49 myotoxin, alone or in combination, upon C2C12 myotubes. The indicated amounts of toxins were added in a total volume of 150 µL. Cytolysis was determined by the release of lactate dehydrogenase to the medium, 3 h after exposure of the cells to the toxins. Each bar represents mean ± SD of triplicate cell cultures. Statistically significant differences (p<0.05) between two groups are indicated by dotted arrow lines.

Since *B. asper* venom also contains non-myotoxic Asp49 PLA_2_s whose role in venom’s toxicity is uncertain [35], the effect of a non-myotoxic, acidic-type PLA_2_ on the cytotoxic activity of Lys49 myotoxin was investigated in the same assay system, as shown in Fig. 7. As expected, the acidic Asp49 enzyme alone was not cytotoxic. The combination of this enzyme with the Lys49 myotoxin caused a significant, although only modest increase at 5 µg, but at 10 µg was unable to significantly enhance cytotoxicity of Lys49 myotoxin (Fig. 7).

**Figure 8:**
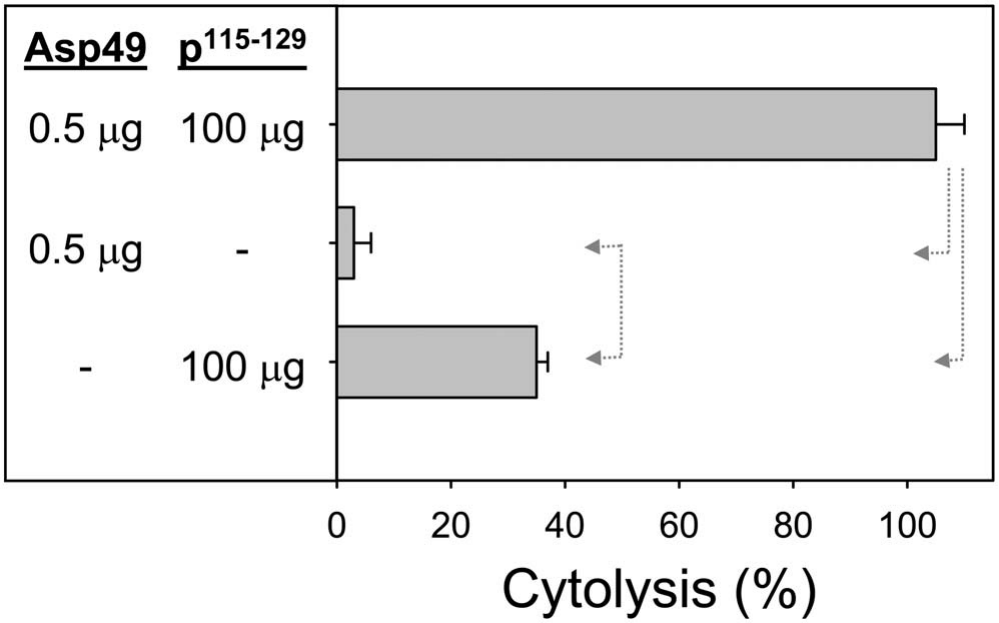
Cytotoxic activity of Asp49 myotoxin and the synthetic C-terminal peptide p115–129 of Lys49 myotoxin II from *Bothrops asper*, alone or in combination, upon C2C12 myotubes. The indicated amounts of toxin or synthetic peptide were added in a total volume of 150 µL. Cytolysis was determined by the release of lactate dehydrogenase to the medium, 3 h after exposure of the cells to the toxins. Each bar represents mean ± SD of triplicate cell cultures. Statistically significant differences (p<0.05) between two groups are indicated by dotted arrow lines.

The synergistic effect of a low amount of Asp49 myotoxin toward the cytolytic action of the synthetic peptide p^115–129^ of the Lys49 myotoxin was evaluated. As presented in Fig. 8, the cytotoxicity induced by this short peptide was markedly enhanced by acting in combination with the Asp49 enzyme.

**Figure 9:**
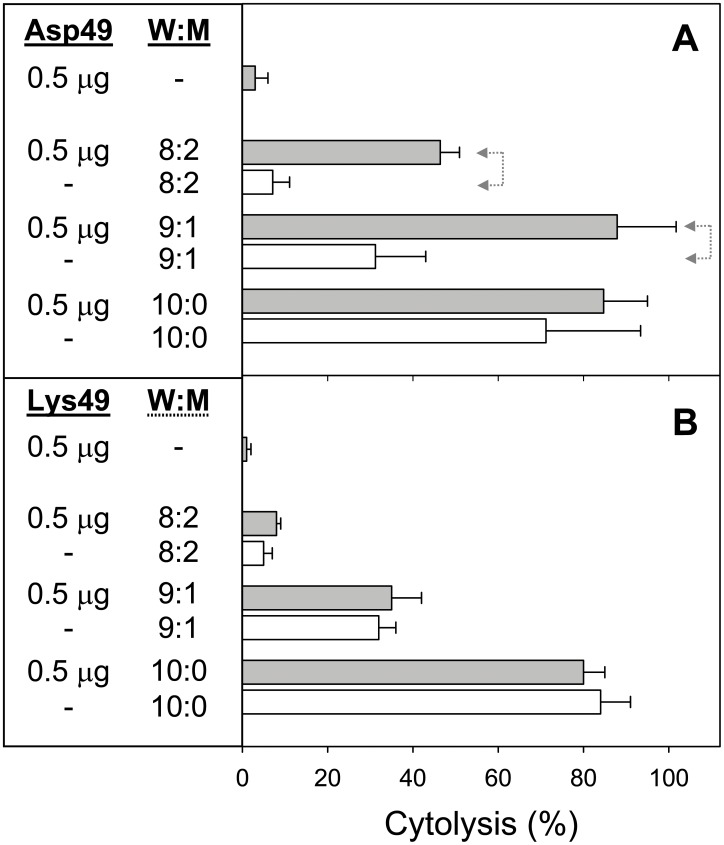
Cytotoxic activity of Asp49 and Lys49 myotoxins upon C2C12 myotubes under conditions of osmotic imbalance. Myotubes were grown and differentiated as described in Methods, and then the toxins (0.5 µg) were added to cultures using medium that contained the indicated proportion of water (gray bars). (**A**) Asp49 myotoxin, (**B**) Lys49 myotoxin. Control cultures exposed to the same medium conditions, in the absence of toxin, were tested in parallel (empty bars). Cytolysis was determined by the release of lactate dehydrogenase to the medium after 3 h. Each bar represents mean ± SD of triplicate cell cultures. Statistically significant differences (p<0.05) between two groups are indicated by dotted arrow lines.

Finally, the cytotoxic action of Asp49 and Lys49 myotoxins was tested under conditions of osmotic imbalance of the cells. Culture medium was rendered hypotonic by the addition of varying proportions of purified water (8∶2, 9∶1, or 10∶1 water:medium), and cytolysis was determined in the absence or presence of the toxins. As shown in Fig. 9A, myotubes exposed to a low amount of Asp49 myotoxin became significantly more susceptible to the deleterious action of the hypotonic media, at 8∶2 and 9∶1 water:medium proportions. At the 10∶0 proportion (100% water), the high cytolysis in the control cells did not allow the assessment of the effect of myotoxin. In contrast, when the same experiment was performed with Lys49 myotoxin, it revealed that this protein does not alter the susceptibility of myotubes exposed to hypotonic conditions, since similar values of cytolysis were observed in the absence or in the presence of the toxin (Fig. 9B).

## Discussion

The venoms of many viperid snake species contain variable combinations of PLA_2_s, often including acidic and basic variants, and among the latter, Asp49 and Lys49 myotoxin subtypes [1], [2], [41]. Phylogenetic analyses indicate that the myotoxic Lys49 PLA_2_ homologues diverged from ancestral, group II Asp49 PLA_2_s before the separation of Viperinae and Crotalinae [42]–[46]. Intriguingly, however, a comprehensive examination of the bioactivities displayed by myotoxic Asp49 and Lys49 variants does not provide evident clues on the possible evolutionary advantages conferred by the emergence of the latter, since both types of myotoxins share similar toxicological profiles and often coexist in viperid venoms [41]. Nevertheless, the abundance and common occurrence of these coexisting myotoxins in many viperid species strongly suggest that they provided an important adaptive value in this family of snakes. Several speculative hypotheses have been proposed to envisage their possible biological significance and adaptive value, one of them being synergism [41]. A synergistic action upon myogenic cells in culture was first described by Cintra-Francischinelli et al. [25] using the C2C12 cell line as a target for Asp49 and Lys49 myotoxins isolated from the venom of *B. asper*. The present study extends such observations by demonstrating the *in vivo* synergism between these two toxin subtypes in the induction of myonecrosis, and provides further insights into the mechanisms of this synergistic effect.

In agreement with previous studies [47], a higher susceptibility of myotubes over myoblasts to the cytotoxic action of the myotoxins was observed. Also, myoblasts were more resistant to the Asp49 than to the Lys49 myotoxin, as previously noted by Cintra-Francischinelli et al. [25]. In agreement with their study, a cytotoxic synergism between the two toxins was confirmed at both stages of cell differentiation, i.e. myoblasts and myotubes. In order to determine whether these *in vitro* observations would also apply to the biologically-relevant target of myotoxins, i.e. skeletal muscle, similar experiments were conducted in mice. Results demonstrate, for the first time *in vivo*, a clear enhancement of myotoxicity by the combined action of Asp49 and Lys49 myotoxins in comparison to the effect of either protein alone. Therefore, these *in vivo* results add new evidence for the adaptive value of the emergence of two subtypes of PLA_2_ myotoxins in viperid venoms, conferring a selective advantage in the light of the high energetic costs of venom protein synthesis [48], [49].

The *in vivo* synergism hereby shown helps to clarify previous observations in the study of viperid PLA_2_ myotoxins, in which crude venoms have generally been found to induce stronger myonecrosis than their isolated myotoxins [50]. Although the contribution to muscle damage of other toxin types in crude venoms (for example hemorrhagic metalloproteinases that promote myonecrosis as a consequence of ischemia [51]) cannot be excluded, the combined action of Asp49 and Lys49 myotoxins in crude venoms may explain the higher magnitude of myonecrosis observed in comparison to experiments analyzing isolated myotoxins. Also noteworthy, the extent of muscle damage induced by the Lys49 myotoxin was higher than that caused by the Asp49 myotoxin. This result is in agreement with the proposal that Lys49 PLA_2_ homologues in viperids provided an adaptive value due to their increased myotoxic potency, as discussed by Kihara et al. [52]. From the biological standpoint, an enhanced capacity to induce acute muscle damage might contribute to a more efficient digestion of the abundant muscle mass characteristic of mammalian prey [41].

In order to determine whether the synergistic mechanism depends on the PLA_2_ activity of Asp49 myotoxins, this enzyme was inactivated by *p*-BPB [22], [24]. As expected, the modified enzyme essentially lost its cytotoxic effect upon myotubes, but surprisingly, still enhanced the cytotoxic action of the Lys49 myotoxin. This prompted us to evaluate the residual catalytic activity of the *p*-BPB-treated protein, which revealed a low, but detectable hydrolysis of the 4-nitro-3-octanoyloxybenzoic acid substrate, estimated at the level of 3% of the unmodified toxin. On this basis, it was hypothesized that such low residual catalytic activity could either be sufficient for the occurrence of synergism, or, alternatively, the synergistic action recorded for the *p*-BPB-modified enzyme would be caused by a catalytically-independent mechanism. To address this point, the synergism was subsequently tested with low amounts of the Asp49 PLA_2_, mimicking the proportion of the corresponding residual enzymatic activity of the *p*-BPB-treated myotoxin. Results confirmed that these minute amounts of the Asp49 PLA_2_, in sublytic concentrations *per se*, are able to enhance the cytolytic effect of the Lys49 myotoxin. Therefore, the mechanism of synergism can be attributed to the enzymatic action of the Asp49 PLA_2_, rather than a catalytically-independent activity. A similar conclusion was reached by Cintra-Francischinelli et al. [25] by observing that, in the absence of external Ca^2+^, the Asp49 myotoxin was unable to synergize with the Lys49 myotoxin due to the requirement of this ion for enzymatic activity. Moreover, the present observations underscore that even a very low enzymatic activity of Asp49 myotoxins is enough for the observed synergism. The directionality of this ‘micro-synergism’ was determined to be an enhancement of the Lys49 myotoxin toxicity by the Asp49 enzyme, and not the converse, since low quantities of the Lys49 myotoxin did not increase the toxic action of the Asp49 enzyme.

Using this experimental model of ‘micro-synergism’, additional aspects of the mechanisms involved were explored. Since the enhancing action of the Asp49 enzyme was found to depend on its catalytic activity, an experiment was designed to evaluate the effect of time by which cells were exposed to the enzyme, then washed, and finally exposed to the Lys49 myotoxin. The addition of a low amount of the Asp49 PLA_2_, independently of the time of contact with the cells, and even when washing was performed immediately after toxin addition, led to a similar cytotoxic outcome. One possibility to explain these findings would be that the enzyme binds rapidly and tightly to the cell membrane interface, and is not removed by gentle washing, thus continuing its enzymatic phospholipid hydrolysis. The assessment of this hypothesis awaits experiments on the binding of myotoxins to myotubes.

Considering that the venom of *B. asper* contains, in addition to basic PLA_2_s, an acidic Asp49 PLA_2_ enzyme which is devoid of myotoxicity (BaspPLA_2_-II [35]), it was of interest to evaluate whether this enzyme would be able to synergize with the basic Lys49 myotoxin. In agreement with its previous characterization, this acidic PLA_2_ did not induce cytotoxicity *per se*. Interestingly, this acidic PLA_2_ did not induce the marked synergistic effect observed with the basic Asp49 PLA_2_. Only a minor increase in cytotoxicity was observed when using 5 µg of the enzyme, and twice this amount did not result in a statistically significant difference of toxicity in comparison to the Lys49 myotoxin alone. This result is noteworthy because the acidic enzyme displays a higher catalytic activity than the Asp49 myotoxin [35]. This suggests that the acidic enzyme might be unable to hydrolyze the membrane phospholipids of myotubes, which in turn would explain both its lack of toxicity and its inability to synergize effectively with Lys49 myotoxin in this model. This hypothesis awaits the study of phospholipid hydrolysis in the membranes of myotubes and muscle cells by using highly sensitive methodologies such as mass spectrometry [26]. Hence, the role of this non-cytotoxic acidic PLA_2_ in the overall toxicity of the venom of *B. asper*, if any, remains uncertain.

A key question arising from the present and previous studies on the synergism between Asp49 PLA_2_ and Lys49 myotoxins concerns how does the enzymatic activity of the former enhance the toxicity of the latter. To the best of our knowledge, the first evidence of a synergism between these two myotoxin subtypes was reported by Shen and Cho [53], who demonstrated that an Asp49 PLA_2_ from *A. p. piscivorus* venom enhanced the liposome-permeabilizing effect of a Lys49 PLA_2_ homologue isolated from the same source. These authors proposed that the Asp49 enzyme would generate anionic patches of hydrolytic reaction products on the surface of the liposomes, which in turn would facilitate the electrostatic interaction with the Lys49 protein, and the consequent permeabilization of the vesicle by the non-enzymatic, bilayer penetrating mechanism of the latter [53]. A second, non-mutually exclusive explanation for the synergistic mechanism was proposed [25], whereby the products of phospholipid hydrolysis generated by the Asp49 myotoxin would enhance the toxicity of the Lys49 myotoxin by rendering the cell membrane more unstable, based on the observation that a mixture of lysophospholipids and fatty acids can mimic *per se* the membrane-damaging effects of Asp49 myotoxin.

**Figure 10:**
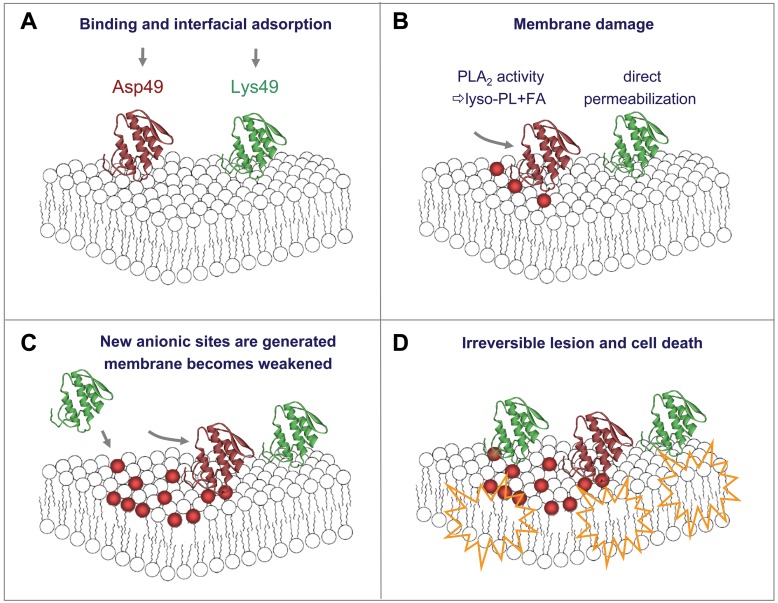
Cartoon representation of the hypothetical synergistic mechanism involved in the membrane-damaging activity of Asp49 and Lys49 myotoxins. Toxins bind to the muscle cell membrane (A), although acceptor moieties for this event are unknown. Each toxin type by its own has the ability to induce cytotoxicity *in vitro* or myonecrosis *in vivo* through membrane damage. Asp49 myotoxins destabilize the membrane by the enzymatic hydrolysis of phospholipids (PL) and consequent production of lyso-PL and fatty acids (FA), whereas Lys49 myotoxins exert a direct permeabilization mechanism via their C-terminal region (B). When acting in combination, FA produced by the Asp49 myotoxin generate new anionic sites (red spheres) that facilitate the binding of Lys49 myotoxin through electrostatic interactions (C). The membrane becomes more unstable due to PL hydrolysis *per se*, and to the accumulation of the reaction products (lysoPL and FA). As a result of these combined actions of Asp49 and Lys49 myotoxins, membrane damage is enhanced and the cell becomes irreversibly damaged (D). Note that the toxin cartoons are represented as monomers for simplicity, although these toxins actually occur as homodimers. Cartoons are not drawn to scale, and the orientation of the toxins interacting with the membrane is only for illustrative purposes.

In the present study, two further observations shed light into the possible mechanisms of the synergy here characterized. First, the bioactive C-terminal synthetic peptide of the Lys49 myotoxin, p^115–129^, reproduced the synergy phenomenon observed with the parent protein, i.e. the concomitant addition of a low amount of Asp49 PLA_2_ and peptide resulted in a significant enhancement of myotube cell lysis. Since this peptide is highly cationic [27], this finding (as well as results with the parent Lys49 myotoxin) would be compatible with the hypothesis that proposed the generation of new anionic sites by the Asp49 enzyme [53], thus facilitating the electrostatic interaction of the peptide or the toxin with the membrane [54], [55], and ultimately its permeabilization [8], [25], [56]. However, the higher cytotoxic effect of the peptide when acting in synergy with the Asp49 myotoxin might as well be explained by the weakening of membrane stability caused by the catalytic action of the enzyme and by the generation of fatty acids and lysophospholipids. Thus, results of this experiment would be compatible with both mechanisms. The hypothesis of a general membrane-destabilizing effect caused by phospholipid hydrolysis and generation of products that alter its biophysical properties [25] is hereby experimentally supported. It was hypothesized that, if the myotube cell membrane becomes more unstable due to the enzymatic action of Asp49 PLA_2_, it would be less capable of resisting a non-specific stress such as osmotic imbalance. Results confirmed this assumption, showing that myotubes were significantly more susceptible to the cytolysis induced by hypotonic media if they were exposed to minute amounts of Asp49 PLA_2_ myotoxin. In contrast, myotubes exposed to equivalent amounts of Lys49 myotoxin were equally susceptible to lysis in such hypotonic media, as in the absence of toxin. Taken together, these experiments indicate that the enhancing mechanism for the toxicity of Lys49 myotoxins exerted by Asp49 PLA_2_ myotoxin involves at least the weakening of cell membrane integrity by the latter. On the other hand, the possibility of the generation of new anionic sites by the accumulation of products of catalysis in the membrane remains to be tested, but clearly, both mechanisms would rely on the enzymatic activity of the Asp49 PLA_2_ myotoxin (Fig. 10).

The synergistic mechanism hereby characterized contributes to rationalize the evolutionary advantage for the emergence of two different subtypes of PLA_2_ myotoxins in the venom of many viperids, which are known to use two contrasting molecular mechanisms that lead to the same outcome: skeletal muscle necrosis. The synergy between Asp49 and Lys49 myotoxins represents at least one advantageous feature for the snakes, but additional mechanisms of adaptive value for these toxins may also exist [41], for example in their possible functional interactions with other snake venom components [57], [58]. On a more general ground, our findings stress the need to study the action of snake venoms from a holistic perspective, i.e. by analyzing not only the action of purified toxins, but also the interaction of different components in the context of the complexity of snakebite envenoming.
